# Effect of flavophospholipol on fecal microbiota in weaned pigs challenged with *Salmonella* Typhimurium

**DOI:** 10.1186/s40813-020-00151-5

**Published:** 2020-05-12

**Authors:** Saranya Nair, Abdolvahab Farzan, J. Scott Weese, Zvonimir Poljak, Robert M. Friendship

**Affiliations:** 1 Department of Population Medicine, Ontario Veterinary College, University of Guelph, Guelph, Ontario Canada; 2 Department of Pathobiology, Ontario Veterinary College, University of Guelph, Guelph, Ontario Canada

## Abstract

**Background:**

The heightened prevalence of *Salmonella* Typhimurium remains a public health and food safety concern. Studies have reported antibiotic, flavophospholipol, may have the ability to reduce *Salmonella* in swine, as well as alter the gut microbiota in favour of beneficial bacteria by inhibiting pathogenic bacteria. Thus, the objective of this study was to investigate the fecal microbiota of weaned pigs receiving in-feed flavophospholipol and challenged with *Salmonella* Typhimurium.

**Results:**

Twenty-one weaned pigs were fed either a diet containing 4 ppm of flavophospholipol (treatment group) or a non-medicated feed (control group) for 36 days post-weaning (Day 1 to Day 36). The pigs were orally challenged with a 2 mL dose of 10^8^ CFU/mL of *S.* Typhimurium at Day 7 and Day 8. Community bacterial DNA extracted from fecal samples collected at Day 6 (before challenge) and Day 36 (28 days after challenge) were used to assess the fecal microbiota using the V4 region of the 16S rRNA gene with Illumina MiSeq next-generation sequencing. Sequencing data were visualized using mothur and analyzed in JMP and R software. The fecal microbiota of pigs in the treatment group had differences in abundance of phyla (Firmicutes, Proteobacteria) and genera (*Lactobacillus, Roseburia*, *Treponema,* unclassified Ruminococcaceae, *Blautia*, *Streptococcus*, *Megasphaera*, *Dorea*, *Sporobacter*, *Peptococcus*, unclassified Firmicutes, *Clostridium* IV and *Campylobacter)* when compared to pigs that were controls, 28 days after challenge with *Salmonella* (*P* < 0.05). Specifically, results demonstrated a significant increase in phylum Proteobacteria (*P =* 0.001) and decrease in Firmicutes (*P* = 0.012) and genus *Roseburia* (*P* = 0.003) in the treated pigs suggestive of possible microbial dysbiosis. An increased abundance of genera *Lactobacillus* (*P* = 0.012) was also noted in the treated group in comparison to the control.

**Conclusion:**

Based on these findings, it is difficult to conclude whether treatment with 4 ppm of flavophospholipol is promoting favorable indigenous bacteria in the pig microbiota as previous literature has suggested.

## Background

With the increased prevalence of non-typhoidal *Salmonella* spp. (*Salmonella*) on swine farms [1, 2], food safety concerns heighten. *Salmonella* Typhimurium, commonly recovered from the feces and tissue of swine [3–5], has been reported worldwide as one of the leading *Salmonella* serotypes causing human enteric illness [6–8]. Pigs may shed *Salmonella* at different stages of production, but in particular *Salmonella* has been found to be prevalent during the nursery or post-weaning stage [9–11]. During this stage, shedding is trigged in pigs, often healthy carriers of *Salmonella*, as a result of stressful events such as transportation, weaning, comingling and change in feed [12].

As pigs grow and develop over time, the porcine intestinal microbiota evolves and changes in composition until a stable bacterial population is established [13]. The immunity in the gut of newly weaned piglets tends to be low as they are no longer receiving easily digestible milk, containing colostrum which comprises immunoglobulins, and are faced with the rapid decline of circulating antibodies [14]. Furthermore, with the introduction of a grain-based diet, a lower feed intake can result in potential disruption to the microbiota and epithelial inflammation [15]. During this stage, as their passive immunity conferred from their dam wanes, they are susceptible to disease and likely to become infected with pathogens (e.g. *Salmonella*) [12, 16]. As the host’s immunity slowly becomes more effective and their microbiota begins to evolve during this time, it is a great opportunity to manipulate their gut health with interventions to help suppress the growth of pathogens like *Salmonella*.

Flavophospholipol (synonyms: moenomycin, flavomycin and bambermycin), a phosphoglycolipid antibiotic produced by *Streptomyces* spp., functions by impairing transglycolase activity of penicillin-binding proteins causing hindrance to the bacterial cell wall synthesis making it primarily effective against Gram-positive bacteria [17–20]. Despite this, studies have reported on the ability of flavophospholipol to reduce *Salmonella* shedding and colonization in swine and poultry [21, 22]. Flavophospholipol may also have the ability to improve the gut microbiota equilibrium by altering the microbial population in favour of beneficial bacteria inhibiting the colonization of pathogenic bacteria (e.g. *Salmonella*) in broiler [21] and tilapia [23]. This inhibitory behaviour by flavophospholipol may be a result of proliferation of beneficial bacteria in competition with pathogenic bacteria for attachment sites on the intestinal epithelium [21, 24]. Flavophospholipol may also indirectly aid in the inhibition of *Salmonella* by the combined increase in production of volatile fatty acids (e.g. acetic, propionic, butyric acids), produced by anaerobic bacteria (e.g. *Lactobacillus*), along with reduced intestinal pH and redox potential [21, 24, 25]. To date, there are limited data available on the influence of flavophospholipol on the fecal microbiota in challenged pigs. Thus, the objective of this study was to evaluate the changes in flavophospholipol treated nursery pig fecal microbiota after challenge with *S.* Typhimurium. The relationship between the fecal microbiota and *Salmonella* status (antibody response, shedding and internal colonization) was also assessed.

## Methods

### Ethics statement

This study was approved by the Animal Care Committee of the University of Guelph, in accordance with the guidelines set forward by the Canadian Council of Animal Care.

### Pigs and sample collection

**Fig. 1:**
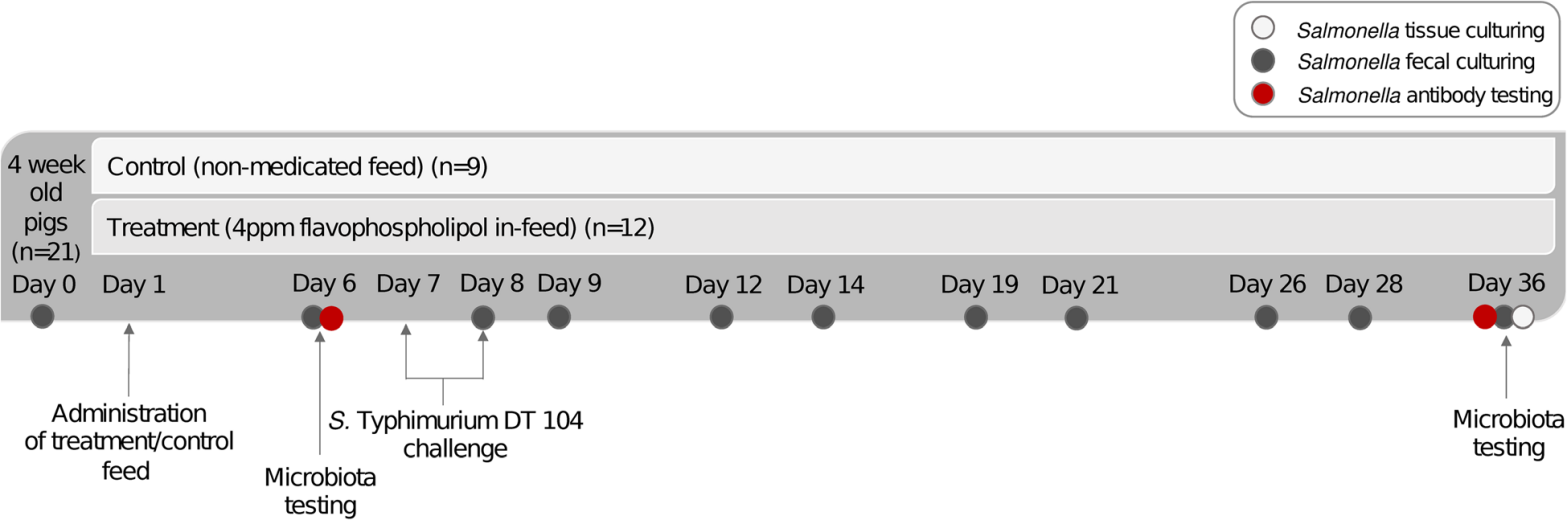
Challenge trial timeline. Figure depicts the study timeline from the arrival of 4-week-old nursery pigs (*n* = 21) on Day 0, treatment with either 4 ppm of flavophospholipol (*n* = 12) or non-medicated control feed (*n* = 9) on Day 1, *Salmonella* Typhimurium DT 104 challenge on Day 7 and 8, to days where *Salmonella* isolation and microbiota testing was conducted over the 36 day trial period

Twenty-one, newly weaned four-week-old, healthy crossbred piglets [(Landrance x Yorkshire) x Duroc] were transferred from the Arkell Swine Research Centre, University of Guelph, to a level 2 biosafety isolation facility at the Ontario Veterinary College, University of Guelph (Day 0). Piglets were randomly assigned to four separate rooms. Two rooms of pigs (*n* = 12) received a medicated diet containing 4 ppm in-feed flavophospholipol (based on recommended dosage; Flavomycin®, Huvepharma, Ontario, Canada), while pigs (*n* = 9) in the other two rooms received an identical diet, without the added medication (Day 1). On Day 7 and 8 of the trial, piglets were orally challenged with a 2 mL dose of 10^8^ colony forming units (CFU)/mL of *S.* Typhimurium DT 104, with resistance to nalidixic acid. Fecal samples were collected on Days 0, 6, and after the challenge on Days 8, 9, 12, 14, 19, 21, 26, 28 and 36. Blood samples were collected on Day 6 and Day 36. At Day 36, the pigs were euthanized and tissue (spleen, liver, ileocecal lymph node) samples were collected. A timeline of the study is illustrated in Fig. 1.

### *Salmonella* isolation and antibody detection

All fecal and tissue samples were cultured for *Salmonella* as previously described [26]. Fecal samples collected on Day 0 and Day 6 determined whether pigs were shedding *Salmonella* prior to challenge, while fecal samples collected on Day 8 determined if the *Salmonella* challenge had infected pigs with *S.* Typhimurium DT 104. Samples collected beyond Day 6, after challenge, were plated on to XLT-4 agar containing nalidixic acid. To determine the *Salmonella* CFU, serial dilutions were made using 1 g of feces diluted with 0.1% buffered peptone water (BPW) (Becton Dickinson™, Sparks Glencoe, Maryland, USA) and plated on XLT-4 agar containing nalidixic acid. All plates were incubated at 37 **°**C for 18 to 24 to 72 h.

Serum samples were assessed for *Salmonella* antibody by an indirect enzyme-linked immunosorbent assay (ELISA) (pigtype® *Salmonella* Ab kit, QIAGEN Leipzig GmbH, Leipzig, Germany) as per kit manual. Using the equation provided by the manufacturer, a sample-to-positive ratio (S/P) value was determined and samples with S/P ratio of ≥ 0.3 were considered *Salmonella* seropositive.

### DNA extraction, 16S rRNA gene PCR amplification and purification

For microbiota processing, fecal samples collected at 2 sampling points over the duration of the trial, at Day 6 (before challenge) and at Day 36 (28 days after challenge) (Fig. 1), stored at − 20 °C, were used.

DNA was extracted from fecal samples using a commercial kit (E.Z.N.A. Stool DNA Kit, Omega Bio-Tek Inc., Doraville, Georgia, USA) following the manufacturer’s protocol. DNA concentration and purity were quantified using spectrophotometry (NanoDrop, Thermo Scientific, USA). The V4 hypervariable region of the 16S rRNA gene was amplified using forward (5′-AYTGGGYDTAAAGNG-3′) and reverse (5′- TACNVGGGTATCTAATCC-3′) primers consisting of overhanging adapter regions to anneal with Illumina universal index sequencing adaptors that were required for a later polymerase chain reaction (PCR) [27]. For the amplification of the 16S rRNA V4 region, a previously describe protocol was used [28]. To assess the quality of the PCR products, electrophoresis using 1.5% agarose gel was used and evaluated under a UV light using the GeneGenius bioimaging system (Syngene, USA). The purification of the PCR product was done using Agencourt AMPure XP beads (Beckman Coulter Inc., Mississauga, Ontario, Canada) following a previously described protocol [28, 29]. Approximately 50 μl of the product was transferred to a microcentrifuge tube and stored at − 20 °C before being processed.

### Indexing, purification and DNA sequencing

To add the Illumina universal adapters to the purified 16S rRNA gene product for indexing, a 50 μl reaction mixture consisting of 25 μl KAPA 2G Fast HotStart ReadyMix 2X, 12 μl of molecular biology-grade water, 4 μl each of the forward and reverse sample-specific Illumina universal adapters with 5 μl of each DNA sample was completed. Previously described conditions were used for a second short PCR cycle which was performed to anneal the index primers to the amplicons [28]. The product was purified with 40 μl AMPure XP beads and DNA was eluted into 35 μl of 10 mM Tris (pH 8.5) buffer. Each sample (32 μl) was transferred to a 96-well plate. The samples (~ 2 μl) were then quantified by spectrophotometry and normalized to a final concentration of 2 nM. To assess the quality of the amplicon library, 5 μL of the sample using electrophoresis with 1.5% agarose gel was used and evaluated under a UV light using the GeneGenius bioimaging system. Sequencing of the amplicon library pool, remaining 25 μL of each sample, was performed using an Illumina MiSeq (San Diego, USA) using 2 × 250 chemistry at the University of Guelph’s Advanced Analysis Centre.

### Analysis of sequencing data and statistical methods

Open-source bioinformatics software package, mothur (v.1.39.5) [30], was used to analyze DNA sequences using the mothur standard operating procedure [31]. Paired-end reads were assembled and aligned to SILVA 16S rRNA reference database to ensure that they were from the 16S rRNA V4 region [32]. Irregularities such as sequence lengths > 245 bp or < 239 bp, ambiguous base calls, and long runs of homopolymers > 8 bp were removed. Sequence data were also screened for chimeras, using UCHIME [33], and non-bacterial domains (chloroplast, mitochondria, Archaea and Eukaryotes) were removed. Remaining sequences were assigned into operational taxonomic units (OTUs) using a de novo (open OTU picking) approach based on a 3.0% dissimilarity threshold.

Relative abundances of the main phyla (median relative abundance > 0.5%) and main genera (inclusion based on high relative abundance) at Day 6 to Day 36 between groups was calculated and presented both graphically and in a chart. Statistical analysis was conducted on main phyla and main genera to assess whether there was any difference between the groups after challenge. Appropriate transformations (i.e. square root, log, inverse) were applied to phyla and genera that were nonparametric to improve normality. A repeated-measures (to account for repeated measures at Day 6 and 36) analysis of variance (ANOVA) model was constructed with JMP 13 (SAS Institute Inc., Cary, NC, USA) for each phylum and genus (dependent variable) with treatment and day of sampling as explanatory variables. Interaction between treatment and day of sampling was also explored. To control for multiple comparisons at the phyla and genera level, the Benjamini & Hochberg’s False Discovery Rate (FDR) [34] analysis using statistical software, R v.3.5.0 (R Foundation for Statistical Computing, Vienna, Austria), was applied to the ANOVA *p*-values. Null hypothesis for all statistical tests was rejected at *P*_FDR_ < 0.05.

Random subsampling of sequences for each sample was conducted to normalize the sequence data. The following alpha diversity measures were calculated using mothur: sampling coverage (Good’s coverage), estimated richness (Chao1 index), evenness (Shannon’s evenness index) and diversity (inverse Simpson index). Using Wilcoxon test, these indices were compared between groups from Day 6 to Day 36, with a statistical significance at *P <* 0.05.

Beta diversity was assessed using Jaccard and Yue & Clayton indices to measure community membership and community structure, respectively. Principle Coordinate Analysis (PCoA), plotted using JMP 13, helped to visualize clustering of groups with these beta diversity indices. Dendrograms, plotted using FigTree v1.4.3, illustrated similarities between groups at the two sampling points for community membership and community structure. Unweighted-Unique Fraction Metric (Unweighted-UNIFRAC), analysis of molecular variance (AMOVA), and homogeneity of molecular variance analysis (HOMOVA) were conducted to evaluate community membership and community structure between groups from Day 6 to Day 36.

Linear discriminant analysis (LDA) effect size (LEfSe) [35] was used to evaluate and identify bacterial taxa that were enriched at the two time points between groups. Inclusion of genera was based on a *P* < 0.05 and LDA score ≥ 2.0. LEfSe results were plotted graphically using JMP 13.

Statistical analysis, using JMP 13, was conducted to evaluate the relationship between the fecal microbiota with *Salmonella* antibody response, *Salmonella* shedding and colonization. The change in *Salmonella* seropositivity, from Day 6 to Day 36, was compared to the change of abundance in main phyla and main genera. A repeated-measures (to account for repeated measures at Day 6 and 36) ANOVA model was generated for each phylum and genus (dependent variable) to assess *Salmonella* seropositivity (explanatory variable). Each model also included treatment, day and possible interactions. The same statistical method was used to assess the change in *Salmonella* antibody titer. This modelling approach was used to see if any of the main phylum or genera had an association with *Salmonella* (seropositivity/antibody response). To control for multiple comparisons at the phyla and genera level, the Benjamini & Hochberg’s FDR analysis was applied to the ANOVA *p*-values. Null hypothesis for all statistical tests was rejected at *P*_FDR_ < 0.05.

For *Salmonella* CFU, statistical analysis was only conducted for main phyla and genera at Day 36 as this was the only time fecal samples were analyzed for microbiota. *Salmonella* CFU (Day 9, 12, 14, 19, 21, 26, 28 and 36; *Salmonella* shedding) [26] was compared to fecal microbiota at Day 36 to assess whether a particular phyla or genera maybe associated with more or less *Salmonella* shedding. A median CFU value was used due to the low range of CFU at Day 36. ANOVA models were constructed for each phylum and genus (dependent variable) modelled with the median CFU as well as treatment. Lastly, *Salmonella* internal colonization, measured based on whether a pig had at least one positive tissue sample at euthanasia, was compared to the nursery pig microbiota at Day 36 to assess whether a particular phyla or genera maybe associated with *Salmonella* colonization. ANOVA models were constructed for each phylum and genus (dependent variable) modelled with tissue. Median CFU and treatment were also included in the model. Benjamini & Hochberg’s FDR was applied to the ANOVA *p*-values for all statistical analysis. Null hypothesis for all statistical tests was rejected at *P*_FDR_ < 0.05.

## Results

The impact of flavophospholipol on *Salmonella* shedding, colonization and antibody response over the duration of this experimental trial has previously been published [26]. The findings revealed medicating the nursery diet with 4 ppm of flavophospholipol was not effective in reducing the presence of *Salmonella* in tissue and feces (*P* > 0.05) [26]. Further, no difference in *Salmonella* antibody response was found between flavophospholipol-treated pigs and non-medicated controls (*P* > 0.05) [26].

Only a subset of fecal samples was available and used to assess the microbiota resulting in an uneven number of treatment and control pigs in the present study. The goal was to identify the microbiota after treatment with flavophospholipol and prior to challenge with *Salmonella* (Day 6) as well as after treatment and challenge (Day 36). Specifically, Day 36 was used because it marks the end of the nursery stage. Earlier time points were not used because of the lack of fecal samples and inconsistencies in sample pairings for pigs. However, these time points allow exploring the change in the fecal microbiota after the nursery period when the microbiota is evolving and can be manipulated with interventions like flavophospholipol.

### *Salmonella* shedding

In the present trial, on Day 0 and 6, before challenge with *S.* Typhimurium, no pigs were identified as shedding *Salmonella* spp. However, following the challenge, all of these pigs were found to be shedding *Salmonella* on Day 8. On Day 36, *Salmonella* was isolated from all pigs, both in the treatment and control group.

### Impact of flavophospholipol on fecal microbiota in pigs challenged with *S.* Typhimurium

#### Sequence quality

The total number of sequences that were recovered from 42 fecal samples on Day 6 (1,557,062) and Day 36 (2,358,324) was 3,915,386. The median number of sequences recovered on Day 6 in the treatment (*n* = 12) and control group (*n* = 9) was 56,086 (Range: 34536, 196,866) and 48,465 (Range: 20213, 171,494), respectively. While, the median number of sequences recovered on Day 36 in the treatment (*n* = 12) and control group (*n* = 9) was 44,448 (Range: 23650, 194,135) and 174,548 (Range: 36333,533,913), respectively. Overall, sequences clustered into 16,382 OTUs, which were classified into 26 bacterial phyla and 639 genera.

#### Relative abundance

**Fig. 2:**
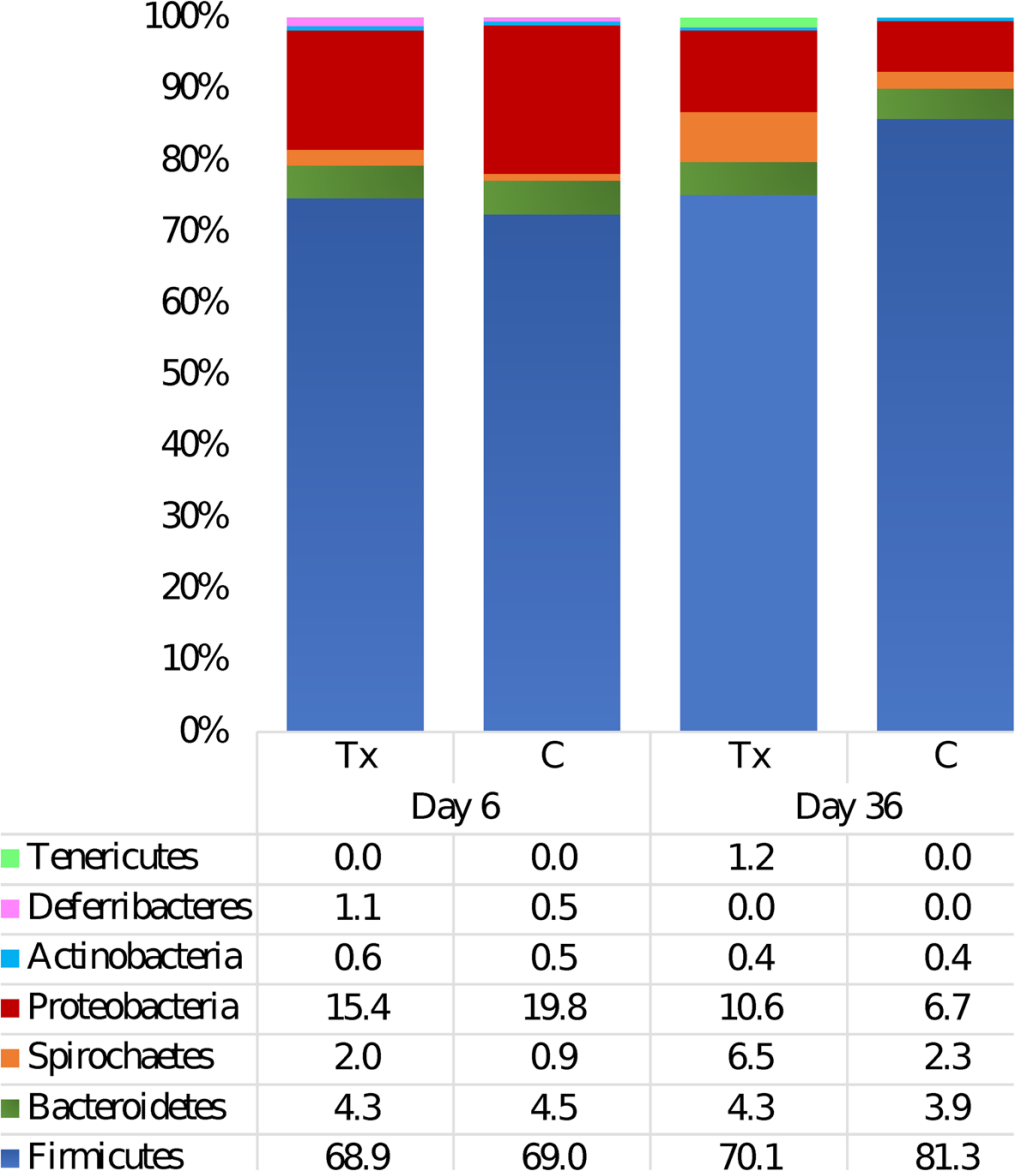
Relative abundance of bacterial phyla after *Salmonella* challenge. Fecal microbiota of 4-week-old pigs at Day 6 (before challenge) and Day 36 (28 days after challenge) treated with either 4 ppm of flavophospholipol (Tx; *n* = 12) or non-medicated control feed (C; *n* = 9) from Day 1 onwards. Figure is limited to phylum that met the > 0.5% median cutoff

Overall, the main phyla were Firmicutes, Bacteroidetes, Spirochaetes, Proteobacteria, Actinobacteria, Deferribacteres and Tenericutes (Fig. 2). From Day 6 to Day 36, Firmicutes, with an interaction between day and treatment in the ANOVA model, were found to increase in both groups but with a larger relative abundance in the controls, which initially had a lower abundance than the treated group (*P*_FDR_ = 0.012) (Additional file 1). Interestingly, Proteobacteria, also with an interaction between day and treatment, started at a greater abundance in the control group and was found to decrease in both groups over time. However, the phylum was found in lower numbers in the control group than the treatment group on Day 36 (*P*_FDR_ = 0.001) (Fig. 2). Meanwhile, the statistical significance found between groups from Day 6 to Day 36 in phyla Deferribacteres, Spirochaetes and Tenericutes was not due to the effect of treatment but was a result of a day effect (Additional file 1).

**Fig. 3:**
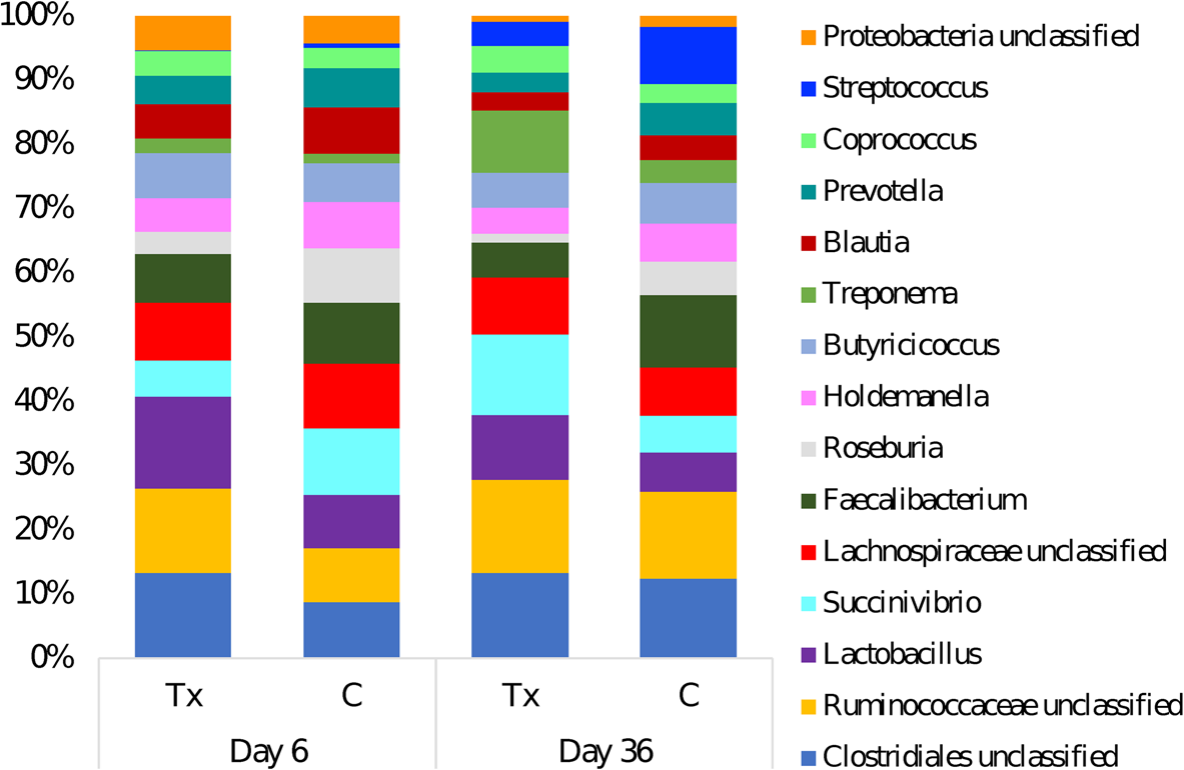
Relative abundance of bacterial genera after *Salmonella* challenge. Fecal microbiota of 4-week-old pigs at Day 6 (before challenge) and Day 36 (28 days after challenge) treated with either 4 ppm of flavophospholipol (Tx; *n* = 12) or non-medicated control feed (C; *n* = 9) from Day 1 onwards. Figure is limited to the top 15 genera

Figure 3 illustrates the relative abundances of the main genera (inclusion of 15 genera based on high relative abundances) at Day 6 and Day 36. The relative abundances along with the *p*-values and FDR *p*-values for a total of the 40 selected genera, including the main genera and an additional 25 genera based on high relative abundance, are presented in Additional file 2. An unclassified Ruminococcaceae, *Roseburia*, *Treponema*, *Blautia*, *Streptococcus*, *Megasphaera*, *Dorea*, *Sporobacter*, unclassified Firmicutes, *Peptococcus*, and *Clostridium* cluster IV were found to be different between groups from Day 6 to Day 36 (*P*_FDR_ < 0.05). Meanwhile, *Lactobacillus* was found in a larger abundance in the treatment group compared to the controls at both Day 6 and Day 36 (*P*_FDR_ = 0.029). *Oscillibacter*, *Ruminococcus*, *Anaerovibrio*, *Escherichia/Shigella*, *Mucispirillum*, *Clostridium* sensu stricto and *Selenomonas* were found to be different at Day 36 from that in Day 6 in both groups. Lastly, *Campylobacter*, with an interaction between day and treatment, was found in greater relative abundance in the control group on Day 6 than the treatment but was found in both groups at similar low levels of abundance on Day 36 (*P*_FDR_ = 0.0001).

Additional file 3 identifies the top 10 dominant genera at Day 6 and Day 36 in the control and treatment group. Over time, unclassified Ruminococcaceae and unclassified Clostridiales are abundant in both groups. *Streptococcus* is also found to be numerous at Day 36 in the treatment group.

#### Alpha and beta diversity

**Fig. 4:**
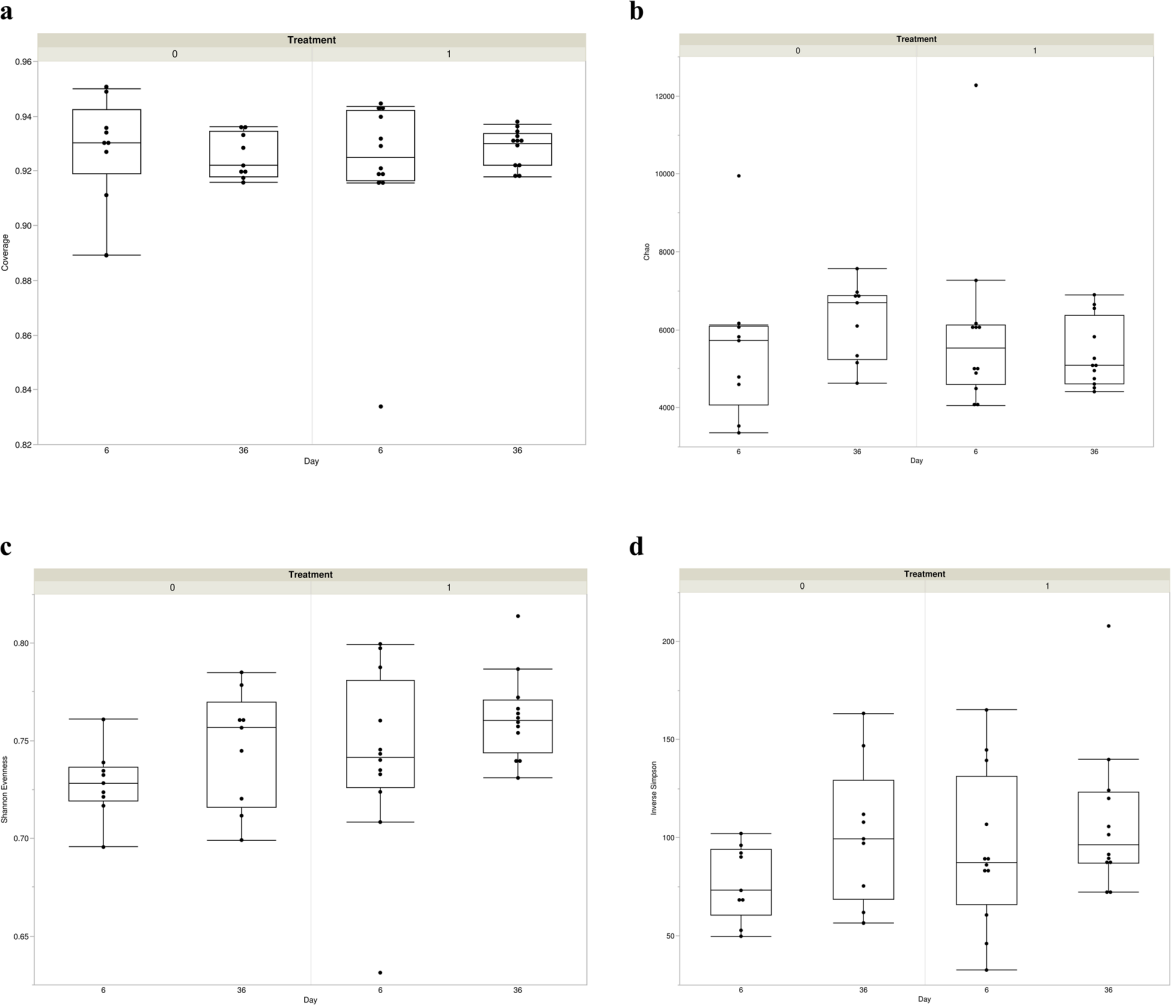
Fecal microbiota alpha diversity after *Salmonella* challenge. Quantile boxplots of (**a**) Good’s Coverage, (**b**) Chao’s Richness, (**c**) Shannon’s Evenness, and (**d**) Inverse Simpson’s Diversity of 4-week-old pigs at Day 6 (before challenge) and Day 36 (28 days after challenge) treated with either 4 ppm of flavophospholipol (Tx; *n* = 12) or non-medicated control feed (C; *n* = 9) from Day 1 onwards

A random sub-sampling of 20,213 sequences for each fecal sample was conducted to normalize samples. Alpha diversity measures, Good’s coverage, Chao’s richness, Shannon evenness and Inverse Simpson diversity, were explored (Fig. 4). From Day 6 to Day 36, no differences in Good’s coverage, Chao’s richness, or Inverse Simpson diversity were noted between the fecal microbiota of nursery pigs fed in-flavophospholipol and pigs fed a non-medicated diet (*P* > 0.05). However, Shannon evenness was found to increase in the flavophospholipol treatment group from Day 6 to Day 36 in comparison to the control group (*P* = 0.046).

**Fig. 5:**
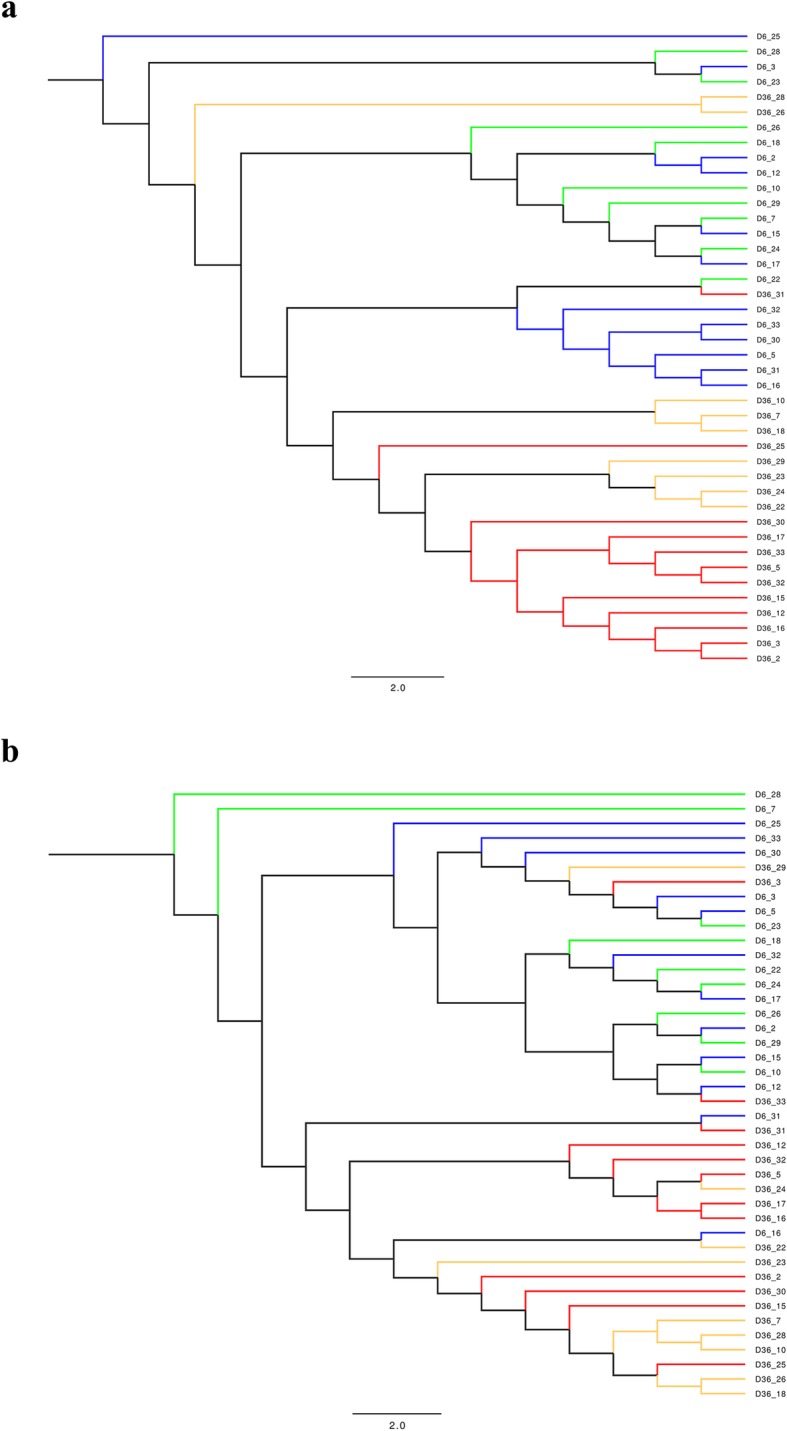
Dendrogram of (**a**) Yue and Clayton index (community structure) and (**b**) Jaccard index (community membership) after *Salmonella* challenge. Based on the fecal microbiota of 4-week-old pigs treated with 4 ppm of flavophospholipol (Tx; *n* = 12) or non-medicated control feed (C; *n* = 9) at Day 6 (before challenge) and Day 36 (28 days after challenge). Treatment on Day 6 (blue), control on Day 6 (green), treatment on Day 36 (red) and control on Day 36 (orange). Treatment was administered from Day 1 onwards

**Fig. 6:**
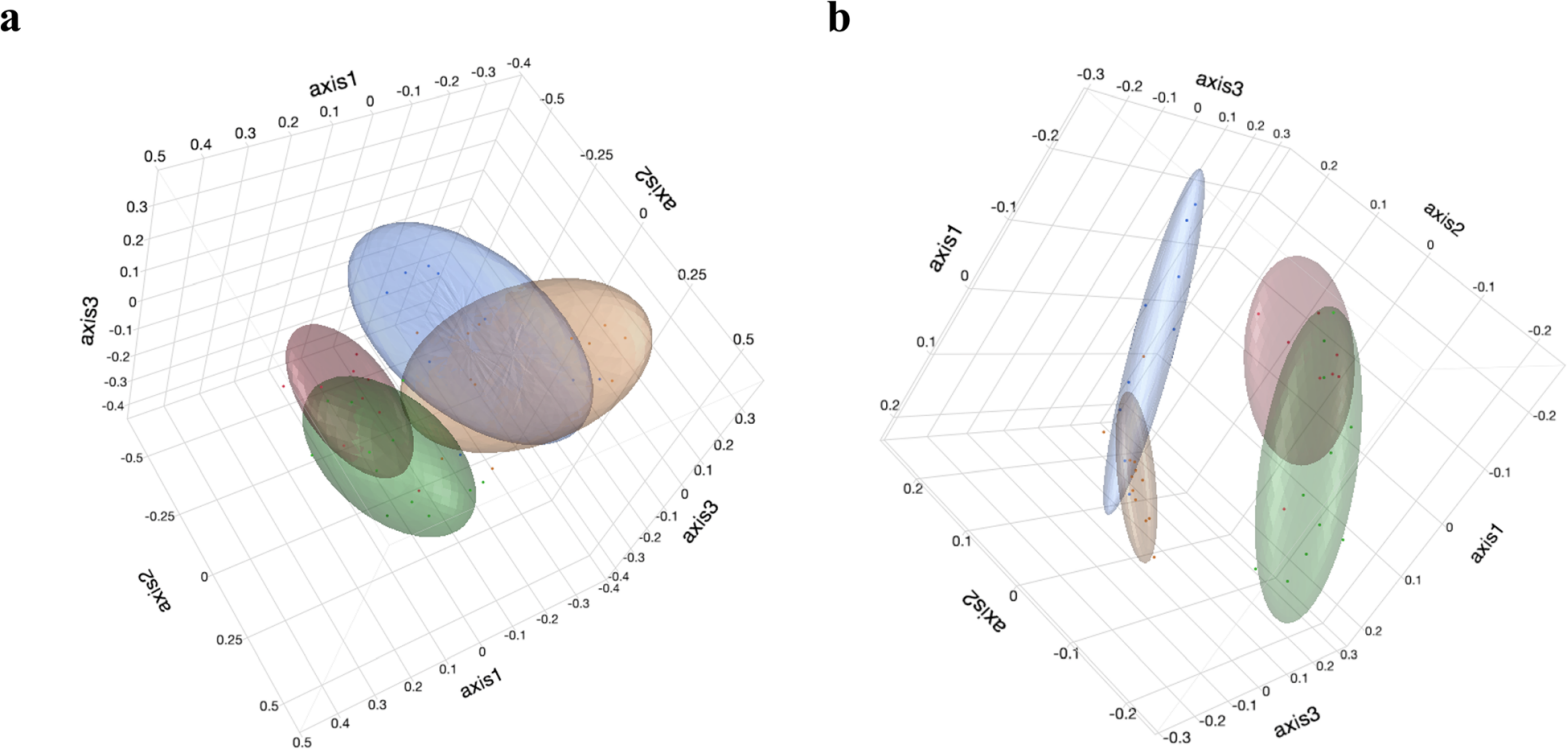
Principal coordinate analysis (PCoA) of (**a**) Yue and Clayton index (community structure) (**b**) Jaccard index (community membership) after *Salmonella* challenge. Based on the fecal microbiota of 4-week-old pigs treated with 4 ppm of flavophospholipol (Tx; *n* = 12) or non-medicated control feed (C; *n* = 9) at Day 6 (before challenge) and Day 36 (28 days after challenge). Treatment on Day 6 (green), control on Day 6 (red), treatment on Day 36 (orange) and control on Day 36 (blue). Treatment was administered from Day 1 onwards

Community membership, measured using the Jaccard index, assessed the similarity between the fecal microbiota of treated pigs in comparison to control pigs based on the ratio of shared taxa to unshared taxa at Day 6 and at Day 36. While, community structure, measured using the Yue & Clayton index, evaluated the structural similarity of the fecal microbiota of treated pigs in comparison to control pigs based on the proportions of the populations that are represented by shared and unshared species. Based on the Jaccard and Yue & Clayton indices, the dendrograms (Fig. 5) illustrates clustering occurring at Day 6 and at Day 36, while the PCoA plots (Fig. 6) reveal clustering between groups at Day 6 and at Day 36. Although, the unweighted UniFrac, AMOVA and HOMOVA tests for both community membership and community structure found a difference between the treatment and control group at Day 6 & Day 36 (*P* < 0.05) (Table 1), the PCoA plots identify some overlap between the groups at the two sampling points.

**Table 1:** Unweighted UniFrac, AMOVA and HOMOVA test values for Jaccard (community membership) and Yue and Clayton (community structure) indices. Based on the fecal microbiota at Day 6 (before challenge) and Day 36 (28 days after challenge) in 4-week-old pigs treated with either 4 ppm of flavophospholipol (Tx; *n* = 12) or non-medicated control feed (C; *n* = 9) from Day 1 onwards

	Unweighted UniFrac	AMOVA	HOMOVA
Day 6 – Day 36			
Jaccard Index	< 0.001	< 0.001	< 0.001
Yue & Clayton Index	0.0010	< 0.001	0.05

**Fig. 7:**
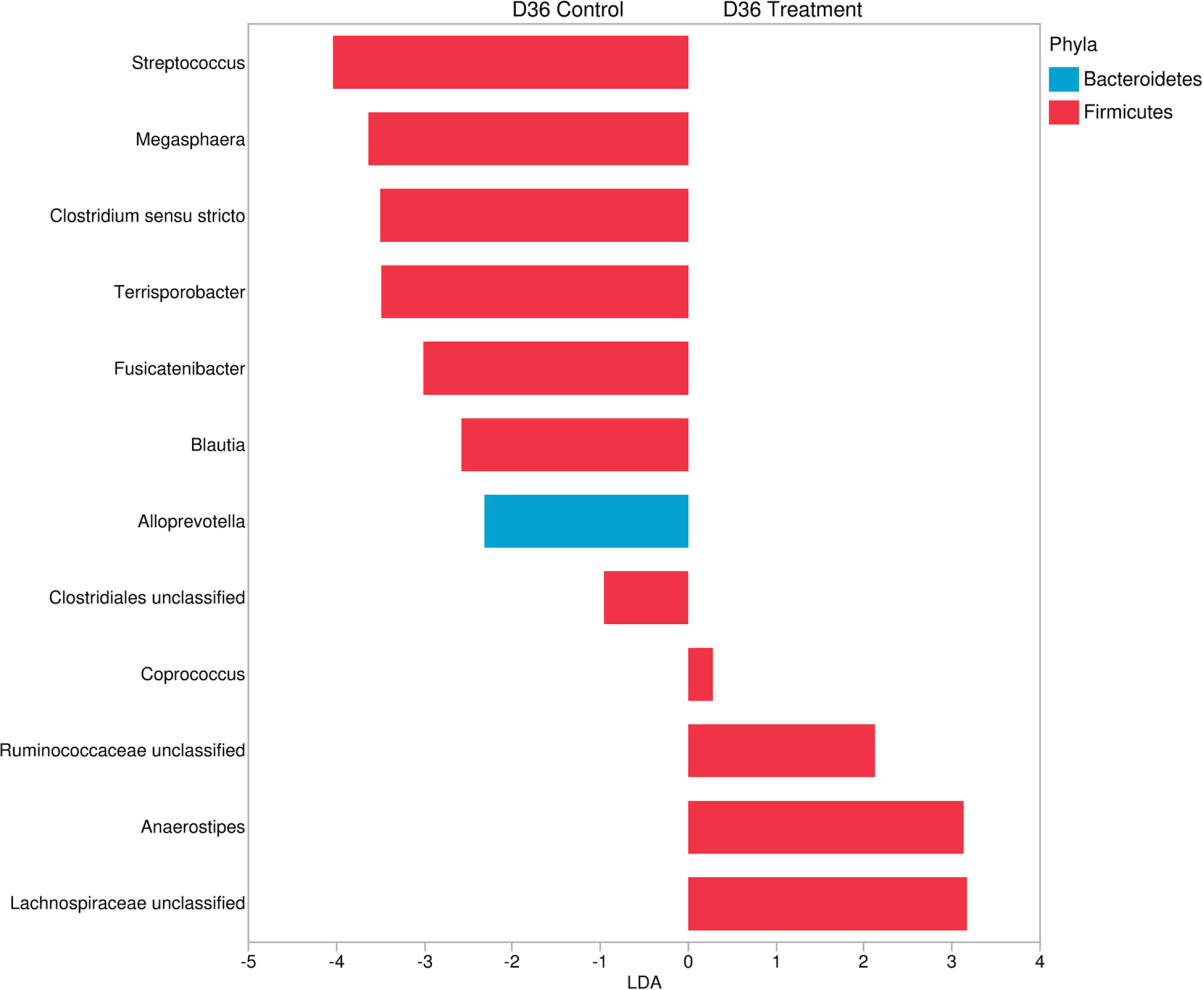
Plot of LEfSe analysis of enriched genera in the treatment group and control group on Day 36. Based on the fecal microbiota of 4-week-old pigs at Day 36 (after *Salmonella* Typhimurium DT 104 challenge on Day 7 and 8) treated with either 4 ppm of flavophospholipol in-feed (Tx; *n* = 12) or non-medicated control feed (C; *n* = 9) from Day 1 onwards

LEfSe was analyzed at Day 6 and Day 36 separately (Additional file 4 and Fig. 7). Genera with an LDA score ≥ 2 and statistical significance (*P* < 0.05) were identified on Day 6 (*n* = 4) and Day 36 (*n* = 12). All the enriched genera on Day 6 were found to belong to phylum Firmicutes. Meanwhile, on Day 36, the enriched genera included Bacteroidetes along with Firmicutes.

### *Salmonella* vs pig fecal microbiota

#### *Salmonella* antibody response

*Salmonella* seropositivity and antibody titer, for the 21 pigs used in this trial is illustrated in Additional file 5. No significant relationship was found between any phylum or genus and *Salmonella* seropositivity or antibody titers in nursery pigs between Day 6 to Day 36 (*P*_FDR_ > 0.05).

#### *Salmonella* CFU and internal colonization

The *Salmonella* CFU for the 21 pigs used in this trial is illustrated in Additional file 6. At Day 36, no relationship was found between the relative abundance of any phylum or genus and *Salmonella* CFU (*P*_FDR_ > 0.05). *Salmonella* internal colonization was found in 3 pigs out of the 21 pigs (14%) euthanized. *Salmonella* was isolated from the lymph node, liver and spleen. At Day 36, no relationship was found between phyla or genera and the quantitation of *Salmonella* at the sampled internal body sites (lymph node, spleen, liver) (*P*_FDR_ > 0.05).

## Discussion

In the present study, the porcine fecal microbiota was found to encompass a rich and diverse bacterial population consistent with previous studies [28, 36]. After the pigs were challenged with *Salmonella*, distinct clustering between the groups for both community structure and membership was noted. This type of clustering between groups with the application of antibiotics has been previously found [37]. Previous research on community membership has also revealed fecal microbiota samples from pigs between 4 to 7 weeks of age tend to cluster into one age dependent category [28]. This study also found community structure to not be significantly different amongst samples from pigs at 5, 6 and 7 weeks of age [28]. This explains why although there are distinctive clusters between treatment and control samples from Day 6 to Day 36, the overlap between time is suggestive of the development of a stable community structure in the present study. In terms of community structure and membership, the application of flavophospholipol may have to occur at an earlier age when the microbiota is more susceptible to change and has not achieved a stable environment.

The relative abundance of bacterial phyla, with predominance of Firmicutes and Proteobacteria, found over the duration of the present study shared similarities in ranges to the findings from previous studies that assessed the fecal microbiota of pigs between similar ages [28, 36]. However, at the end of the present trial, flavophospholipol-treated pigs showed a lower relative abundance of Firmucutes and greater Proteobacteria than control group. The increased presence of Proteobacteria has been widely reported in different diseases and different species, as an indicator of disease or dysbiosis [38, 39]. Swine research suggests the dysbiosis associated with increased abundance of Proteobacteria may result in diarrhea in nursery pigs [40]. The expansion of Proteobacteria has been found to be associated with gastrointestinal inflammation and the development of colitis in mice [41]. In addition, gut microbial dysbiosis in pigs was associated with increased abundance of Proteobacteria linked to porcine epidemic diarrhea virus (PEDV) [42].

Interestingly, the increase in Proteobacteria in flavophospholipol treated pigs noted in the present study has been previously documented in the fecal microbiota of newly weaned pigs treated with antibiotic treatment, ASP250 (a combination of chlortetracycline 100 g/ton, sulfamethazine 100 g/ton and penicillin 50 g/ton) [43]. Subsequently, with the increase in Proteobacteria in the ASP250 treated pigs, there was an increase in populations of *Escherichia coli*, belonging to the Enterobacteriaceae family [43]. In mice, the use of antibiotics has also been found to result in the disruption of the microbiota composition with increased levels of Proteobacteria as well as inflammation in the intestines [44, 45]. Based on these previous findings, the use of the antibiotic flavophospholipol, in the present study may have a disruptive impact on the porcine fecal microbiota with the expansion of Proteobacteria. This is contrary to previous research suggesting flavophospholipol may have the ability to alter the microbiota in favour of beneficial bacteria while inhibiting opportunistic bacteria [21, 23].

Parallel to the increase of Proteobacteria, a lower abundance of Firmicutes have also been associated with IBD (inflammatory bowel disease) and dysbiosis [46]. In the present study, there was a depletion of *Roseburia,* a Gram-positive short-chain fatty acid (SCFA) producing genus belonging to phylum Firmicutes, in flavophospholipol-treated pigs. *Roseburia* has been reported as one of the most proficient butyrate, a SCFA, producers in the gut microbiota of both human and pig [47, 48]. Ranging from anti-inflammatory properties to possible therapeutic strategies for disease prevention and treatment, the benefits of butyrate, a crucial energy source for colonic epithelial cells, has been well documented [47, 49]. A recent study found the reduced production of *Roseburia hominis* was associated with dysbiosis and pathogenesis of IBD ulcerative colitis in humans [48]. Although previous research has discussed the ability of flavophospholipol to potentially aid in improving the gut microbiota equilibrium [21, 23], the increased abundance of Proteobacteria along with a lower abundance of Firmicutes and genus *Roseburia* in treated pigs reveals possible adverse effects associated with antibiotic treatment that maybe a sign of dysbiosis in the nursery pig fecal microbiota.

In the present study, a greater abundance of *Lactobacillus* was found in the flavophospholipol treated group. Interestingly, a previous study assessing the impact of 2 ppm of flavophospholipol in-feed on the gut microbiota of broiler chick in the first 17 days posthatch found a reduced prevalence of *Lactobacillus* compared to the control microbiota [50]. The difference in the presence of *Lactobacillus,* between the previous study and the present study, is likely a result of variations in dosage of flavophospholipol, duration of treatment and differences in species. However, the increased presence of *Lactobacillus* in flavophospholipol treated pigs in the present study maybe an indication of improved swine health and growth performance with genus’ probiotic attributes like low pH, resistance to bile salts, adhesion to host epithelium, antimicrobial and immunomodulatory properties, as well as competitive exclusion of pathogens [51–55]. Although *Lactobacillus* is praised for its probiotic attributes, there is a lack of supporting evidence on its specific role as it can vary based on the *Lactobacillus* species and the host breed [51, 52]. In a recent study, the administration of *L. salivarius* in pigs was found to not improve growth performance [56]. Whereas, *L. gasseri* has been found to elicit many of the probiotic attributes listed earlier, however in humans [51]. Thus, whether or not the increased presence of *Lactobacillus* in flavophospholipol treated pigs is favourable is debatable and would require species level analysis.

Bacteria with possible beneficial or commensal traits were also found in abundance in the control group. *Blautia*, a Gram-positive bacterium belonging to phylum Firmicutes, was found enriched in the control porcine fecal microbiota after challenge. This genus, which includes key anaerobic intestinal commensal organisms [57], has been associated with anti-inflammatory properties [58, 59]. *Megasphaera*, an obligate anaerobic Gram-negative bacterium belonging to phylum Firmicutes, was also found to be enriched in the control microbiota. *Megasphaera* is a SCFA-producing genus, with the ability to produce amino acids and vitamins [60]. Meanwhile, *Streptococcus*, a Gram-positive genus which includes opportunistic species (e.g. *Streptococcus suis)*, was found to be enriched in controls pigs. Previous research has found with the dominance in *S. suis* in the intestines of post-weaned pigs*,* along with a reduced abundance of *Lactobacillus*, this may result in the impairment of the defensive barrier of the stomach [61]. Although, in the present study, a reduced abundance of *Lactobacillus* is found in the control group, it is difficult to confer whether treatment is having a positive impact on the gut microbiota, by keeping opportunistic *Streptococcus* species at bay, without being able to identify at a species level.

Based on these findings, there is an abundance of certain beneficial/commensal bacteria along with the possible presence of some opportunistic bacteria in both the flavophospholipol-treated pigs and control pigs after *S.* Typhimurium DT 104 challenge. It is difficult to confer from these findings whether the treatment with flavophospholipol, primarily effective against Gram-positive bacteria, can help to improve the porcine intestinal microbiota for the overall health of the pig or in defense against *Salmonella* as suggested in literature.

It is also important to note that along with the antibiotic intervention, the intestinal microbiota of these pigs was impacted by the stress of weaning and change in diet. Previous studies have reported on the significant shift in composition and diversity of microbiota profiles as a result of stress of weaning and change in diet from a highly digestible milk to a less digestible solid feed [36, 62]. Meanwhile, a recent study found that challenge with wild type *S.* Typhimurium induced inflammation in the porcine intestinal gut tissue resulting in a decrease of indigenous bacterial population [63]. This resulted in a reduction in colonization resistance eventually leading to the host being susceptible to *Salmonella* colonization [63]. Thus, when assessing the impact of flavophospholipol on the porcine fecal microbiota, the microbial dysbiosis that occurs with weaning conditions and the challenge with *Salmonella* should be taken into consideration.

The *Salmonella* shedding (CFU) and seropositivity between pigs in flavophospholipol-treated and control groups was similar as published previously [26]. Although, several studies have found differences in gut bacterial population in the low and high *Salmonella* shedder pigs as well as in the colonization of tissue post-*Salmonella* challenge [63, 64], the present study found no associations between the pig fecal microbiota and *Salmonella* CFU or *Salmonella* internal colonization. This is likely because of the lack of variation in *Salmonella* CFU between pigs and due to the low number of pigs with *Salmonella* internal colonization [26]. In addition, if the microbiota was assessed at earlier timepoints, soon after challenge, the impact of *Salmonella* on the microbial populations might have been characterized better. It is likely that the lack of association found between *Salmonella* antibody response and the fecal microbiota was also due to the lack of variation in *Salmonella* antibody response amongst pigs [26]. Other components like biomarkers would have to be considered in order to establish an association between *Salmonella* antibody response and pig fecal microbiota.

This exploratory study sought to identify the differences in fecal microbiota between the flavophospholipol-treated and the not-treated control nursery pigs before and after *Salmonella* challenge. This study also evaluated the relationship between the fecal microbiota and *Salmonella* antibody response, shedding and internal colonization in nursery pigs. One limitation of this study is that the microbiota was only tested once post challenge, at Day 36. However, testing at multiple time points after challenge could have allowed for a better understanding of the evolution of the nursery pig fecal microbiota with the flavophospholipol intervention. Also, testing fecal microbiota at earlier time points might have improved the characterization of the fecal microbiota with regards to *Salmonella*. Although all pigs were shedding *Salmonella* at the end of the nursery stage, earlier testing might have revealed the microbial dysbiosis caused by *Salmonella*. In addition, not assessing growth performance, diarrhea scores, histopathology, and biomarkers presents a limitation when reporting and discussing the outcome of *Salmonella*. The inclusion of these components in future studies could result in a better understanding of the fecal microbiota with *Salmonella* challenge.

Future research with a larger sample size exploring different dosages, frequencies and durations of flavophospholipol is warranted. In addition, flavophospholipol is a commonly used an antimicrobial growth promoter in livestock it is important to consider its impact on growth performance while exploring its impact the gut or fecal microbiota. The presence of a secondary control group that did not receive medicated feed or *Salmonella* challenge, a limitation in the present study, could have been beneficial to assess the baseline porcine fecal microbiota. Lastly, it is important to note that the fecal microbiota cannot be extrapolated to the entire gastrointestinal tract as it is only representative of the distal portion (colon and cecum).

## Conclusions

The porcine fecal microbiota of nursery pigs treated with 4 ppm of flavophospholipol in-feed or a non-medicated feed were found to have variations in microbial populations before and after challenge with *S.* Typhimurium DT 104. Significant differences in potentially beneficial and opportunistic bacteria in both the treatment and control group were found. However, the increased abundance of phylum Proteobacteria and decreased abundance of Firmicutes and *Roseburia* noted in flavophospholipol treated pigs is suggestive of microbial dysbiosis in nursery pigs. Further research with a larger sample size, testing the microbiota at multiple timepoints, including a secondary control group (without treatment or challenge) and exploring different dosages and frequencies is warranted to draw conclusions on the impact of flavophospholipol on the fecal microbiota and gut health. Lastly, no associations were found between the pig fecal microbiota and *Salmonella* CFU, *Salmonella* internal colonization or *Salmonella* antibody response.

## Supplementary information

**Additional file 1. **Intestinal microbiota phyla after challenge (percent relative abundances, *p*-values, and FDR *p*-values). 4-week-old pigs at Day 6 (before challenge) and Day 36 (after challenge) treated with either 4 ppm of flavophospholipol (Tx; *n* = 12) or non-medicated control feed (C; *n* = 9) from Day 1 onwards. Figure is limited to phylum that met the > 0.5% median cutoff.

**Additional file 2. **Intestinal microbiota genera after challenge (percent relative abundances, *p*-values, and FDR p-values). 4-week-old pigs at Day 6 (before challenge) and Day 36 (after challenge) treated with either 4 ppm of flavophospholipol (Tx; *n* = 12) or non-medicated control feed (C; *n* = 9) from Day 1 onwards. Limited to selected 40 genera.

**Additional file 3. **Relative abundance of the top ten dominant genera after challenge. Intestinal microbiota of flavophospholipol treated pigs (Tx; *n* = 12) or non-medicated control pigs (C; *n* = 9) at Day 6 (before challenge) and Day 36 (after challenge). Treatment was administered from Day 1 onwards.

**Additional file 4. **Plot of LEfSe analysis of enriched genera in the treatment group and control group on Day 6. Based on the intestinal microbiota of 4-week-old pigs at Day 6 treated with either 4 ppm of flavophospholipol in-feed (Tx; *n* = 12) or non-medicated control feed (C; *n* = 9) from Day 1 onwards.

**Additional file 5. ***Salmonella* antibody response in subset of pigs used over the duration of the trial. Based on the seropositivity and antibody tiers of nursery pigs, fed a medicated diet with 4 ppm flavophospholipol (*n* = 12) or a control (non-medicated) diet (*n* = 9) for the duration of the trial from Day 1 to Day 36. Pigs were also challenged orally with *Salmonella* Typhimurium DT 104 on Day 7 and 8.

**Additional file 6. ***Salmonella* shedding in subset of pigs used over the duration of the trial. Based on *Salmonella* colony forming unit (CFU)/g of feces in nursery pigs, fed a medicated diet with 4 ppm flavophospholipol (*n* = 12) or a control (non-medicated) diet (*n* = 9) for the duration of the trial from Day 1 to Day 36. Pigs were also challenged orally with *Salmonella* Typhimurium DT 104 on Day 7 and 8.

## Data Availability

The datasets supporting the conclusions of this article are available upon request of the corresponding author.
