# Supplementary material for: Effect of flavophospholipol on fecal microbiota in weaned pigs challenged with Salmonella Typhimurium
Source: Porcine Health Manag. 2020 May 12;6:14. doi: 10.1186/s40813-020-00151-5 (PMC7216395; doi:10.1186/s40813-020-00151-5)
Supplement: Supplementary file 1 — Additional file 1. Intestinal microbiota phyla after challenge (percent relative abundances, p-values, and FDR p-values). 4-week-old pigs at Day 6 (before challenge) and Day 36 (after challenge) treated with either 4 ppm of flavophospholipol (Tx; n = 12) or non-medicated control feed (C; n = 9) from Day 1 onwards. Figure is limited to phylum that met the > 0.5% median cutoff. [file 40813_2020_151_MOESM1_ESM.docx]

**Additional file 1. Intestinal microbiota phyla after challenge (percent relative abundances, p-values, and FDR p-values).** 4-week-old pigs at Day 6 (before challenge) and Day 36 (after challenge) treated with either 4 ppm of flavophospholipol (Tx; n=12) or non-medicated control feed (C; n=9) from Day 1 onwards. Figure is limited to phylum that met the >0.5% median cutoff.

|  | Median % (Min, Max) | | | | *P*-value | *P*_FDR_ |
| --- | --- | --- | --- | --- | --- | --- |
|  | Day 6 | | Day 36 | |  |  |
|  | Tx (n=12) | C  (n=9) | Tx (n=12) | C  (n=9) |  |  |
| Firmicutes^ab^ | 68.9 | 69.0 | 70.1 | 81.3 | 0.009 | 0.012 |
| Proteobacteria^ab^ | 15.4 | 19.8 | 10.6 | 6.7 | 0.000 | 0.001 |
| Bacteroidetes | 4.3 | 4.5 | 4.3 | 3.9 | 0.999 | 0.999 |
| Spirochaetes^b^ | 2.0 | 0.9 | 6.5 | 2.3 | 0.000 | 0.000 |
| Bacteria unclassified | 2.9 | 1.8 | 2.3 | 2.4 |  |  |
| Actinobacteria | 0.6 | 0.5 | 0.4 | 0.4 | 0.200 | 0.233 |
| Deferribacteres^b^ | 1.1 | 0.5 | 0.0 | 0.0 | 0.000 | 0.000 |
| Tenericutes^b^ | 0.0 | 0.0 | 1.2 | 0.0 | 0.004 | 0.008 |

^a^Significiant interaction between day and treatment

^b^Significance effect of day

^c^Significance effect of treatment
