# Supplementary material for: Effect of flavophospholipol on fecal microbiota in weaned pigs challenged with Salmonella Typhimurium
Source: Porcine Health Manag. 2020 May 12;6:14. doi: 10.1186/s40813-020-00151-5 (PMC7216395; doi:10.1186/s40813-020-00151-5)
Supplement: Supplementary file 2 — Additional file 2. Intestinal microbiota genera after challenge (percent relative abundances, p-values, and FDR p-values). 4-week-old pigs at Day 6 (before challenge) and Day 36 (after challenge) treated with either 4 ppm of flavophospholipol (Tx; n = 12) or non-medicated control feed (C; n = 9) from Day 1 onwards. Limited to selected 40 genera. [file 40813_2020_151_MOESM2_ESM.docx]

**Additional file 2. Intestinal microbiota genera after challenge (percent relative abundances, p-values, and FDR p-values).** 4-week-old pigs at Day 6 (before challenge) and Day 36 (after challenge) treated with either 4 ppm of flavophospholipol (Tx; n=12) or non-medicated control feed (C; n=9) from Day 1 onwards. Limited to selected 40 genera.

|  | Median % (Min, Max) | | | | *P*-value | *P*_FDR_ |
| --- | --- | --- | --- | --- | --- | --- |
|  | Day 6 | | Day 36 | |  |  |
|  | Tx  (n=12) | C  (n=9) | Tx  (n=12) | C  (n=9) |  |  |
| Clostridiales unclassified^bc^ | 7.3 | 4.8 | 8.8 | 7.7 | 0.035 | 0.064 |
| Ruminococcaceae unclassified^bc^ | 7.2 | 4.6 | 9.6 | 8.5 | 0.002 | 0.004 |
| Lactobacillus^c^ | 7.8 | 4.6 | 6.7 | 3.7 | 0.014 | 0.029 |
| Succinivibrio | 3.1 | 5.6 | 8.3 | 3.6 | 0.754 | 0.770 |
| Lachnospiraceae unclassified^b^ | 4.9 | 5.6 | 5.9 | 4.7 | 0.683 | 0.712 |
| Faecalibacterium | 4.1 | 5.2 | 3.6 | 7.1 | 0.099 | 0.145 |
| Roseburia^bc^ | 1.9 | 4.7 | 0.9 | 3.2 | 0.001 | 0.003 |
| Holdemanella | 2.9 | 4.0 | 2.7 | 3.7 | 0.227 | 0.291 |
| Butyricicoccus | 3.8 | 3.3 | 3.6 | 4.0 | 0.856 | 0.856 |
| Treponema^bc^ | 1.2 | 0.8 | 6.4 | 2.2 | <0.0001 | 0.000 |
| Blautia^bc^ | 3.0 | 4.0 | 1.8 | 2.4 | <0.0001 | 0.000 |
| Prevotella | 2.4 | 3.4 | 2.0 | 3.2 | 0.271 | 0.308 |
| Coprococcus | 2.1 | 1.7 | 2.7 | 1.8 | 0.494 | 0.537 |
| Streptococcus^bc^ | 0.1 | 0.3 | 2.5 | 5.6 | <0.0001 | 0.000 |
| Proteobacteria unclassified | 2.9 | 2.4 | 0.6 | 1.1 | 0.063 | 0.104 |
| Firmicutes unclassified^abc^ | 1.6 | 0.5 | 1.4 | 1.7 | 0.003 | 0.007 |
| Oscillibacter^b^ | 1.0 | 1.2 | 1.9 | 1.7 | <0.0001 | 0.000 |
| Megasphaera^c^ | 0.2 | 0.1 | 0.1 | 2.0 | 0.026 | 0.049 |
| Phascolarctobacterium | 1.3 | 0.8 | 1.6 | 1.2 | 0.058 | 0.099 |
| Ruminococcus^b^ | 0.9 | 0.6 | 1.7 | 1.4 | 0.004 | 0.009 |
| Gemmiger | 0.7 | 0.8 | 1.0 | 2.0 | 0.069 | 0.111 |
| Dorea^c^ | 0.5 | 1.3 | 0.4 | 0.9 | <0.0001 | 0.000 |
| Bulleidia | 1.2 | 0.7 | 1.4 | 1.3 | 0.184 | 0.243 |
| Ruminococcus2 | 0.9 | 1.4 | 0.5 | 1.1 | 0.102 | 0.146 |
| Erysipelotrichaceae unclassified | 1.0 | 0.4 | 0.9 | 0.6 | 0.234 | 0.293 |
| Propionispira | 0.4 | 0.6 | 0.4 | 0.6 | 0.286 | 0.318 |
| Anaerovibrio^b^ | 0.1 | 0.1 | 0.6 | 1.3 | <0.0001 | 0.000 |
| Anaeroplasma | 0.0 | 0.0 | 1.2 | 0.0 | 0.156 | 0.211 |
| Sporobacter^bc^ | 0.5 | 0.2 | 1.1 | 0.8 | 0.001 | 0.002 |
| Anaerostipes | 0.4 | 0.4 | 0.7 | 0.6 | 0.094 | 0.143 |
| Escherichia/Shigella^b^ | 0.9 | 1.0 | 0.1 | 0.1 | 0.000 | 0.001 |
| Campylobacter^ab^ | 0.4 | 1.3 | 0.3 | 0.0 | <0.0001 | 0.000 |
| Clostridium_IV^bc^ | 0.4 | 0.2 | 0.7 | 0.5 | 0.000 | 0.001 |
| Mucispirillum^b^ | 1.1 | 0.5 | 0.0 | 0.0 | <0.0001 | 0.000 |
| Hungatella | 0.7 | 0.4 | 0.1 | 0.3 | 0.083 | 0.129 |
| Clostridium sensu stricto^b^ | 0.1 | 0.1 | 0.2 | 0.4 | 0.000 | 0.001 |
| Clostridium_XI | 0.2 | 0.4 | 0.5 | 0.3 | 0.264 | 0.308 |
| Peptococcus^abc^ | 0.1 | 0.0 | 0.4 | 0.9 | <0.0001 | 0.000 |
| Selenomonas^b^ | 0.3 | 0.5 | 0.0 | 0.0 | <0.0001 | 0.000 |
| Alphaproteobacteria unclassified | 0.1 | 0.1 | 0.3 | 0.3 | 0.524 | 0.558 |

^a^Significiant interaction between day and treatment

^b^Significance effect of day

^c^Significance effect of treatment
