# Supplementary material for: Effect of flavophospholipol on fecal microbiota in weaned pigs challenged with Salmonella Typhimurium
Source: Porcine Health Manag. 2020 May 12;6:14. doi: 10.1186/s40813-020-00151-5 (PMC7216395; doi:10.1186/s40813-020-00151-5)
Supplement: Supplementary file 3 — Additional file 3. Relative abundance of the top ten dominant genera after challenge. Intestinal microbiota of flavophospholipol treated pigs (Tx; n = 12) or non-medicated control pigs (C; n = 9) at Day 6 (before challenge) and Day 36 (after challenge). Treatment was administered from Day 1 onwards. [file 40813_2020_151_MOESM3_ESM.docx]

**Additional file 3. Relative abundance of the top ten dominant genera after challenge**. Intestinal microbiota of flavophospholipol treated pig (Tx; n=12) or non-medicated control pig (C; n=9) at Day 6 (before challenge) and Day 36 (after challenge). Treatment was administered from Day 1 onwards.

|  | Dominant genera  Median relative abundance (%), (Min, Max) | |
| --- | --- | --- |
|  | Treatment (n=12) | Control (n=9) |
| Day 6 | *Clostridiales* unclassified  7.3 (0.99, 16.5)  *Ruminococcaceae* unclassified  7.2 (2.3, 11.7)  *Lactobacillus*  7.8 (4.2, 13.3)  *Succinivibrio*  3.1 (1.6, 12.6)  *Lachnospiraceae* unclassified  4.9 (2.0, 9.2)  *Faecalibacterium*  4.1 (1.4, 8.3)  *Roseburia*  1.9 (.3, 14.2)  *Holdemanella*  2.9 (1.2, 23)  *Butyricicoccus*  3.8 (1.1, 6.6)  *Treponema*  1.2 (0, 6.9) | *Succinivibrio*  5.6 (1.2, 16.7)  *Lachnospiraceae* unclassified  5.6 (1.7, 7.6)  *Faecalibacterium*  5.2 (2.8, 11.9)  *Clostridiales* unclassified  4.8 (1.9, 7.7)  *Roseburia*  4.7 (2.3, 8)  *Ruminococcaceae* unclassified  4.6 (1.3, 6.5)  *Lactobacillus*  4.6 (2.7, 12.1)  *Holdemanella*  4.0 (1.1, 8.5)  *Blautia*  4.0 (3.2, 6.8)  *Prevotella*  3.4 (1.6, 8) |
| Day 36 | *Ruminococcaceae* unclassified  9.6 (5.3, 15)  *Clostridiales* unclassified  8.8 (4.9, 10.3)  *Succinivibrio*  8.3 (1.9, 14.8)  *Lactobacillus* 6.7 (5.2, 9.2)  *Treponema*  6.4 (1.2, 19.7)  *Lachnospiraceae* unclassified  5.9 (3.9, 11.2)  *Butyricicoccus*  3.6 (2.3, 4.6)  *Faecalibacterium*  3.6 (1.4, 6)  *Coprococcus*  2.7 (1.3, 5.2)  *Holdemanella*  2.7 (1, 6) | *Ruminococcaceae* unclassified  8.5 (3, 14.3)  *Clostridiales* unclassified  7.7 (2.9, 11.8)  *Faecalibacterium*  7.1 (1.8, 9.7)  *Streptococcus*  5.6 (0.6, 8.2)  *Lachnospiraceae* unclassified  4.7 (3.3, 9.5)  *Butyricicoccus*  4.0 (2.2, 5.2)  *Lactobacillus*  3.7 (0.7, 9.8)  *Holdemanella*  3.7 (1.0, 7.1)  *Succinivibrio*  3.6 (1.9, 9.8)  *Roseburia*  3.2 (0.6, 7.7) |
