# Supplementary material for: Effect of flavophospholipol on fecal microbiota in weaned pigs challenged with Salmonella Typhimurium
Source: Porcine Health Manag. 2020 May 12;6:14. doi: 10.1186/s40813-020-00151-5 (PMC7216395; doi:10.1186/s40813-020-00151-5)

**Additional file 4. Plot of LEfSe analysis of enriched genera in the treatment group and control group on Day 6.** Based on the intestinal microbiota of 4-week-old pigs at Day 6 treated with either 4 ppm of flavophospholipol in-feed (Tx; n=12) or non-medicated control feed (C; n=9) from Day 1 onwards.


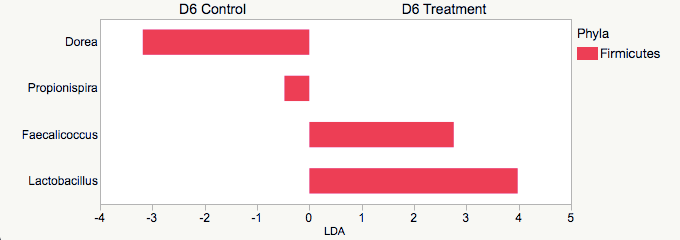

Supplement: Supplementary file 4 — Additional file 4. Plot of LEfSe analysis of enriched genera in the treatment group and control group on Day 6. Based on the intestinal microbiota of 4-week-old pigs at Day 6 treated with either 4 ppm of flavophospholipol in-feed (Tx; n = 12) or non-medicated control feed (C; n = 9) from Day 1 onwards. [file 40813_2020_151_MOESM4_ESM.docx]
