# Supplementary material for: Effect of flavophospholipol on fecal microbiota in weaned pigs challenged with Salmonella Typhimurium
Source: Porcine Health Manag. 2020 May 12;6:14. doi: 10.1186/s40813-020-00151-5 (PMC7216395; doi:10.1186/s40813-020-00151-5)
Supplement: Supplementary file 6 — Additional file 6. Salmonella shedding in subset of pigs used over the duration of the trial. Based on Salmonella colony forming unit (CFU)/g of feces in nursery pigs, fed a medicated diet with 4 ppm flavophospholipol (n = 12) or a control (non-medicated) diet (n = 9) for the duration of the trial from Day 1 to Day 36. Pigs were also challenged orally with Salmonella Typhimurium DT 104 on Day 7 and 8. [file 40813_2020_151_MOESM6_ESM.docx]

**Additional file 6. *Salmonella* shedding in subset of pigs used over the duration of the trial.** Based on *Salmonella* colony forming unit (CFU)/g of feces in nursery pigs, fed a medicated diet with 4 ppm flavophospholipol (n=12) or a control (non-medicated) diet (n=9) for the duration of the trial from Day 1 to Day 36. Pigs were also challenged orally with *Salmonella* Typhimurium DT 104 on Day 7 and 8.
